# Risk of Clinically Relevant Venous Thromboembolism in Critically Ill Patients With COVID-19: A Systematic Review and Meta-Analysis

**DOI:** 10.3389/fmed.2021.647917

**Published:** 2021-03-09

**Authors:** Johannes Gratz, Marion Wiegele, Mathias Maleczek, Harald Herkner, Herbert Schöchl, Eva Chwala, Paul Knöbl, Eva Schaden

**Affiliations:** ^1^Department of Anesthesiology, Intensive Care Medicine and Pain Medicine, Medical University of Vienna, Vienna, Austria; ^2^Department of Emergency Medicine, Medical University of Vienna, Vienna, Austria; ^3^Department of Anaesthesiology and Intensive Care Medicine, AUVA Trauma Centre Salzburg, Academic Teaching Hospital of the Paracelsus Medical University, Salzburg, Austria; ^4^Ludwig Boltzmann Institute for Experimental and Clinical Traumatology, AUVA Trauma Research Centre, Vienna, Austria; ^5^University Library, Medical University of Vienna, Vienna, Austria; ^6^Division of Hematology and Hemostasis, Department of Medicine I, Medical University of Vienna, Vienna, Austria

**Keywords:** venous thromboembolism, COVID-19, incidence, pulmonary embolism, deep vein thrombosis, critically ill patients

## Abstract

**Background:** Early during the course of the ongoing COVID-19 pandemic, reports suggested alarmingly high incidences for thromboembolic events in critically ill patients with COVID-19. However, the clinical relevance of these events was not reported in several studies. Additionally, more recent research showed contradictory results and suggested substantially lower rates of venous thromboembolism. Thus, the aim of the present study was to summarize evidence on the incidence of clinically relevant venous thromboembolism (VTE)—defined as VTE excluding isolated subsegmental pulmonary embolism (PE) and distal deep vein thrombosis (DVT)—in adult critically ill patients with COVID-19.

**Methods:** We performed a systematic review of studies reporting the incidence of clinically relevant PE and/or DVT in critically ill patients with COVID-19. Scientific reports published in the English language between January and October 2020 were included. We conducted a random-effects model meta-analysis to calculate incidence estimates of clinically relevant VTE and bleeding events. We also performed exploratory meta-regression and subgroup analyses of different diagnostic approaches and additional factors that possibly influenced the incidence of these outcomes.

**Results:** Fifty-four articles (5,400 patients) fulfilled the predefined inclusion criteria, of which 41 had a high risk of bias. The majority of included patients were male, > 60 years, and overweight. Twenty-one studies reported the use of prophylactic doses of heparin. Pooled incidences for clinically relevant PE were estimated at 8% (95% CI, 4–11%), for proximal DVT at 14% (95% CI, 9–20%), and—after exclusion of studies with a high risk of bias—for the composite outcome of VTE at 18% (95% CI, 13–24%). Clinically relevant bleeding occurred at a rate of 6% (95% CI, 2–9%).

**Conclusions:** We summarized currently available data on the rate of clinically relevant VTE in critically ill patients with COVID-19. Pooled incidence estimates were lower than those reported by previous review articles. In the absence of evidence-based anticoagulation guidelines for critically ill patients with COVID-19, the results of our study provide clinically important information for an individual risk-benefit assessment in this context.

**Registration:** The study protocol was prospectively registered in PROSPERO on June 22, 2020 (CRD42020193353; https://www.crd.york.ac.uk/prospero).

## Introduction

The COVID-19 pandemic has spread globally since the beginning of 2020, with ~74.5 million confirmed cases and >1.6 million deaths worldwide as of December 21, 2020 (1). SARS-CoV-2 infection has been linked to a wide spectrum of clinical presentations, ranging from mild courses to critical illness (2). A number of publications have indicated that in a subset of patients with COVID-19, coagulopathy could complicate the course of disease and might have an impact on mortality (3–5). For critically ill patients with COVID-19 in particular, early reports suggested an alarmingly high incidence of thromboembolic events of up to 69% (6). However, these numbers have been contradicted by more recent publications, reporting radiographically confirmed venous thromboembolism (VTE) in 8% of critically ill patients with COVID-19 (7).

It has been decades since VTE—defined as the occurrence of deep vein thrombosis (DVT) or pulmonary embolism (PE)—was recognized as a common and potentially fatal complication in critically ill patients (8). Accordingly, current guidelines strongly recommend the use of pharmacological thromboprophylaxis for all critically ill patients without contraindications (9–11).

Hence, it was not surprising when, early in the course of the pandemic, Tang et al. reported a decrease in mortality in patients with COVID-19 with the use of anticoagulant treatment (12). Meanwhile, a number of interim guidance documents on the coagulation management of hospitalized patients with COVID-19 have emerged. Some authors recommend the use of high-prophylactic doses of heparin (13), whereas others suggest that higher doses should be considered in critically ill patients (14), although the results of large-scale clinical trials comparing the use of different anticoagulant regimens in critically ill patients with COVID-19 are still pending (15). However, bleeding has been identified as a relevant risk in critically ill patients (16), and the use of higher doses of antithrombotic agents might further aggravate this risk. Recent publications highlight that the ideal dose of anticoagulants still remains unclear (17).

To better understand these conflicting data and to inform evidence-based guidelines for clinicians, it is important to assess reliable data on the incidence of *clinically relevant* VTE and of bleeding episodes in patients with COVID-19. Thus, the aim of this systematic review was to provide robust estimates of clinically relevant VTE incidence rates in adult critically ill patients with COVID-19 together with estimates of bleeding rates.

## Methods

This systematic review was conducted in accordance with the Preferred Reporting Items for Systematic Reviews and Meta-Analyses (PRISMA) guidelines (18). The study protocol was prospectively registered in PROSPERO (CRD42020193353).

### Literature Search and Study Selection

MEDLINE (via OVID), Embase, Cochrane Central Register of Controlled Trials (CENTRAL), and Web of Science were searched by a dedicated librarian (EC) to identify studies published between January 1, 2020, and October 7, 2020. The detailed search strategy is provided in Additional File 1. In addition, the bibliographies of the included articles were searched by hand.

After deduplication of the search results, titles and abstracts were screened in duplicate for potential relevance by two independent investigators (JG, MW). Interventional and retrospective or prospective observational studies reporting the incidence of radiographically confirmed VTE (i.e., DVT and/or PE) in adult critically ill patients with COVID-19 were included. Studies reporting VTE rates in preselected patient cohorts undergoing specific diagnostic procedures rather than a collective of critically ill patients were excluded. Similarly, postmortem studies were excluded. Furthermore, reports in any language other than English were excluded. Publications judged to be potentially relevant underwent a full-text assessment to determine inclusion by two independent investigators (JG, MW). Disagreements on study eligibility were resolved by consensus or adjudication by a third investigator (ES).

### Data Extraction and Outcomes

Data were extracted into a predefined form in duplicate by two independent investigators (JG, MW). Disagreements were resolved by consensus or adjudication by a third investigator (ES). Extracted data included (i) study details (e.g., study design, publication date, institutional review board (IRB) approval), (ii) patient characteristics (e.g., number of included patients, age, body mass index), (iii) predefined outcomes (e.g., DVT, PE, overall VTE rate), and (iv) potential confounders (e.g., active cancer, duration of disease, type of anticoagulation).

***Primary outcomes***of interest were the incidence of (i) clinically relevant and radiographically confirmed PE and (ii) clinically relevant and radiographically confirmed DVT. We judged PE to be clinically relevant when the deterioration of patients' conditions close to the time of diagnosis was reported (e.g., abrupt hemodynamic and/or respiratory deterioration led to a radiographic examination confirming the diagnosis of PE). Additionally—and specifically if details of the patients' conditions were lacking—we subtracted the number of reported cases of isolated subsegmental PE from the overall number of reported cases of PE. With regard to DVT, as an approximation of clinical relevance, we subtracted the number of reported cases of isolated distal DVT from the overall number of reported cases of DVT. With the same intent, we performed a subgroup analysis according to whether routine ultrasound screening was performed to detect DVT. We did not include catheter-related thrombosis in the definition of DVT.

***Secondary outcomes***included the overall number of any form of PE, the overall number of any form of DVT, and the composite outcome of any form of VTE. Furthermore, the rate of clinically relevant bleeding events (including intracranial bleeding as a subcategory) was determined.

Additionally, we extracted the number of computed tomography (CT) scans performed. Type of anticoagulation was categorized as none, standard (= high-risk prophylaxis) dose heparin, high-dose heparin, any dose heparin, or other forms of anticoagulation. We did not differentiate between the use of low-molecular-weight heparin or unfractioned heparin because the majority of studies did not provide this information.

### Quality Assessment

Currently, there is no available standardized risk of bias assessment tool for incidence or prevalence studies (19). Therefore, we evaluated three different tools in a pilot examination of five studies performed by two independent investigators (JG, MW): the tool developed by Hoy et al. the Joann Briggs Institute Critical Appraisal Checklist for Prevalence Studies, and ROBINS-i (19–21). The tool developed by Hoy et al. was found to have the highest interrater reliability and was thus subsequently used for the quality assessment of the included studies. Briefly, it focuses on five factors determining external validity and five factors determining internal validity using 10 questions. When applicable, questions covering internal validity were answered separately for the outcomes of PE and DVT. When the lack of details provided in a study prevented the answering of a question, the respective item was determined to have a high risk of bias. A final summary item for the overall risk of bias identified studies as having a low, moderate, or high risk of bias. Quality assessment was performed in duplicate by two independent investigators (JG, MW), and disagreements were resolved by consensus or adjudication by a third investigator (HH). The risk of bias assessment was performed only with regard to the relevant outcomes for the present meta-analysis and did not judge the overall quality of included studies.

### Statistical Analysis

We conducted a random-effects model meta-analysis to calculate pooled estimate incidence rates and 95% confidence intervals (CI 95%) for the following four predefined outcomes: (i) PE, (ii) DVT, (iii) VTE, and (iv) bleeding episodes. We corrected for clinically relevant types of PE by calculating the pooled incidence rates of non-subsegmental PE. Similarly, we corrected for clinically relevant types of DVT by calculating the pooled incidence rates of proximal DVT. Additionally, we performed exploratory random-effects meta-regression and subgroup analyses for a number of factors that possibly influenced the estimated incidence rates, including (i) different diagnostic approaches (ultrasound screening for DVT, proportion of patients undergoing CT scans), (ii) quality of the included studies, (iii) date of publication, (iv) sample size of included studies, and (v) different anticoagulation regimens. The heterogeneity of included trials is reported using *I*^2^. To account for small-study effects, zero-event studies were not included in the main analysis. However, a sensitivity analysis was carried out that included zero-event studies using a mixed-effects model to calculate pooled estimate incidences and 95% CI. For each investigated outcome, forest plots were produced. Each meta-regression was visualized using bubble plots. Microsoft Excel (Microsoft, Redmond, WA, USA), Python (Version 3), and Stata (Version 16, College Station, TX, USA) were used for data management, statistical analyses, and graph production.

## Results

### Study and Patient Characteristics

**Figure 1:**
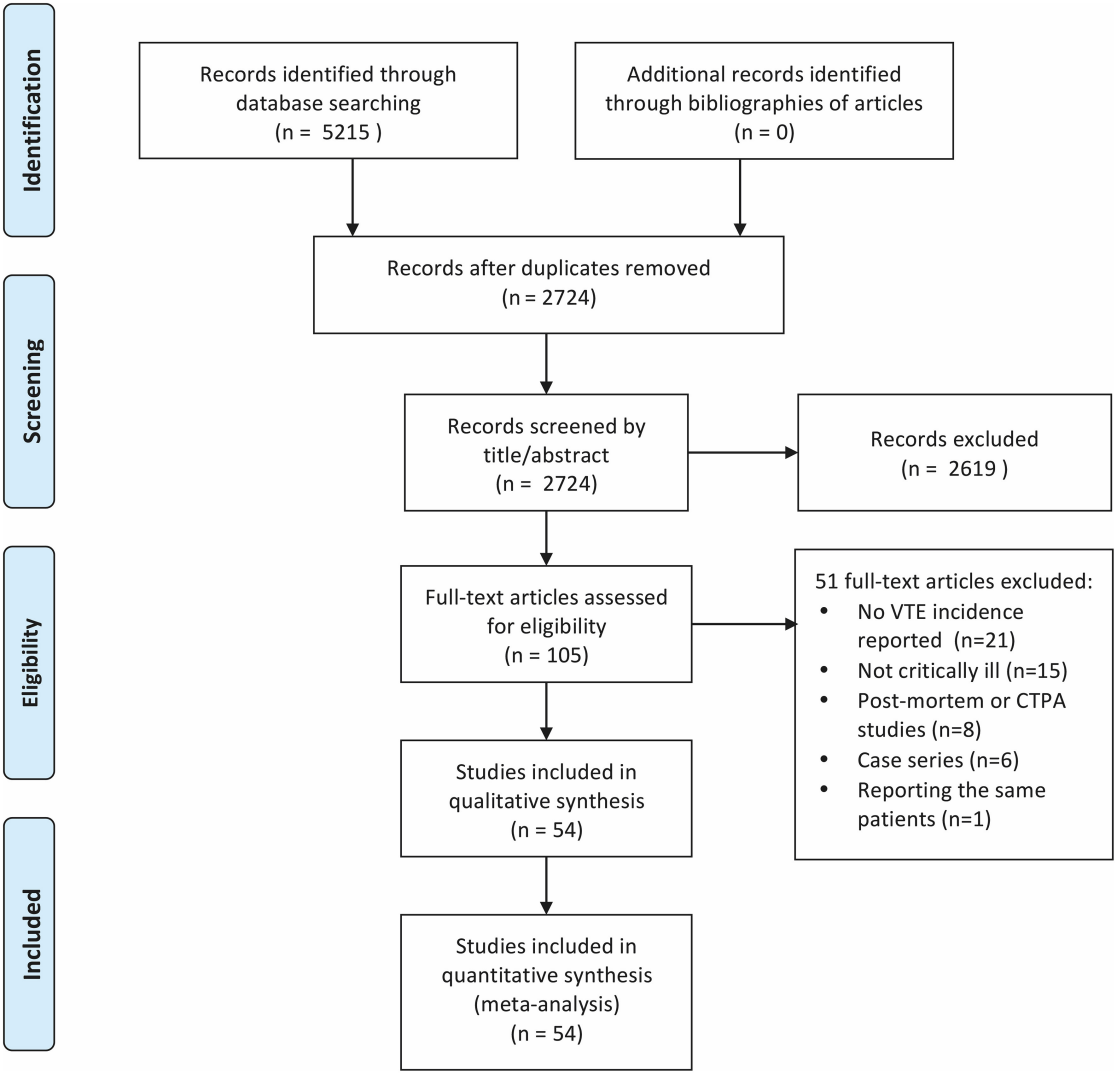
PRISMA flowchart showing the process from identification to inclusion of articles.

The literature search yielded 5,215 results, of which 54 were deemed eligible for inclusion (6, 7, 22–73). Figure 1 presents the process used for the identification, screening, and inclusion of articles.

The included studies reported on a total of 5,400 critically ill patients with COVID-19 from four different continents (Asia, Europe, North America, South America). Detailed characteristics of the studies are shown in Supplementary Table 1. The majority of the included studies reported retrospectively collected data, whereas two studies were prospective, interventional trials. Of note, four studies did not report having obtained IRB approval, and two studies explicitly stated not having sought IRB approval. The number of included patients per study ranged from 16 to 829 patients. Sample sizes for the extracted outcomes—the denominators—ranged from 1,074 patients (secondary outcome of intracranial bleeding) to 5,400 patients (composite outcome of VTE). Regarding the quality assessment, 41 of the studies were found to have a high risk of bias, whereas 13 studies were deemed to carry a moderate risk of bias. None of the included studies were judged to have a low risk of bias with regard to reporting the relevant outcomes. Supplementary Table 2 shows the detailed results of the quality assessment of the included studies.

Supplementary Table 3 shows the relevant patient characteristics, including possible confounders regarding VTE incidence, as well as the thromboprophylactic or anticoagulant regimen for each included study. A substantial number of studies did not report relevant confounding parameters, such as age, body mass index (BMI), length of stay in the intensive care unit (ICU LOS), or disease duration. Studies that reported detailed patient characteristics included a largely comparable patient collective. The overall trend in these studies was that the majority of patients were male, with an advanced age > 60 years, and overweight. Thirteen studies reported an average BMI ≥ 30 kg/m^2^. With regard to thromboprophylaxis and anticoagulation, 21 studies reported using a prophylactic standard heparin dose, whereas 24 studies reported the use of mixed or higher doses of heparins. Five studies reported using other anticoagulant substances, one study explicitly reported not having used thromboprophylaxis at all, and three studies did not provide details about the form of anticoagulation.

### Primary Outcomes: Clinically Relevant PE and DVT

**Figure 2:**
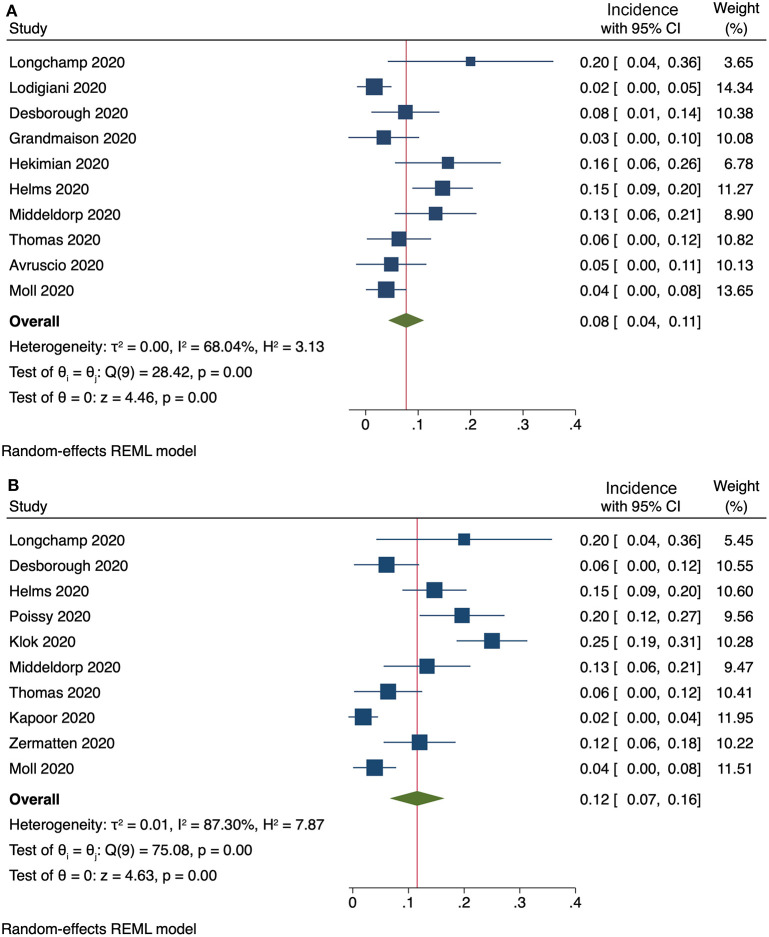
**(A)** Incidence of clinically relevant PE (10 studies) and **(B)** incidence of non-subsegmental PE (10 studies).

Ten studies provided enough information to extract data on the occurrence of ***clinically relevant PE***. The pooled incidence of clinically relevant PE was 8% (95% CI, 4–11%), with a substantial heterogeneity among studies (*I*^2^ = 68%, Figure 2A). Exclusion of subsegmental forms of PE was possible in ten studies, resulting in a pooled incidence of 12% (95% CI, 7–16%) for non-subsegmental PE and a considerable heterogeneity among studies (*I*^2^ = 87%, Figure 2B).

**Figure 3:**
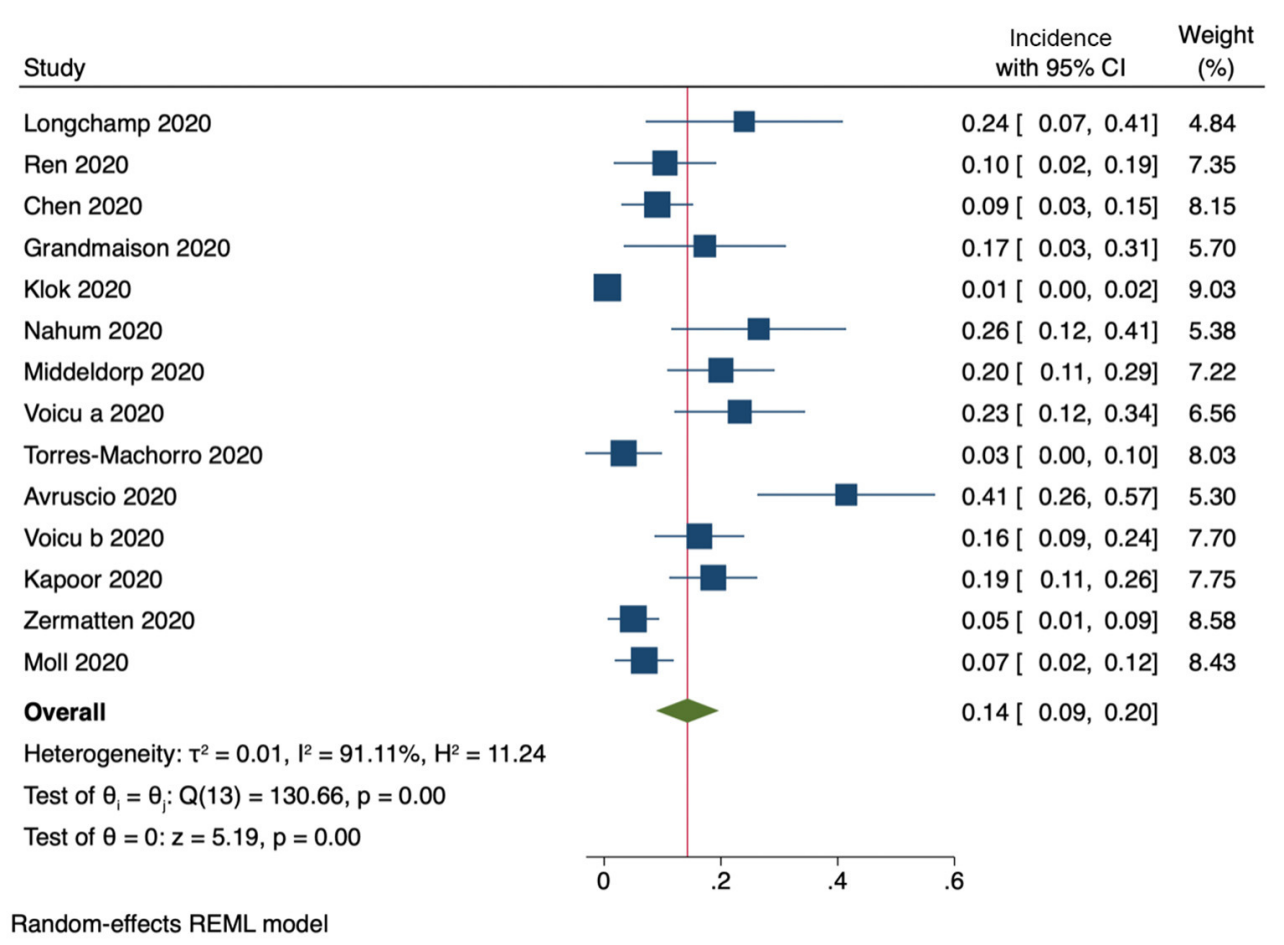
Incidence of proximal DVT (14 studies).

Fourteen studies provided enough information to subtract cases of isolated distal DVT from the total number of reported cases of DVT. The pooled incidence of ***proximal DVT***was 14% (95% CI, 9–20%, Figure 3). Heterogeneity among studies was considerably high (*I*^2^ = 91%).

**Figure 4:**
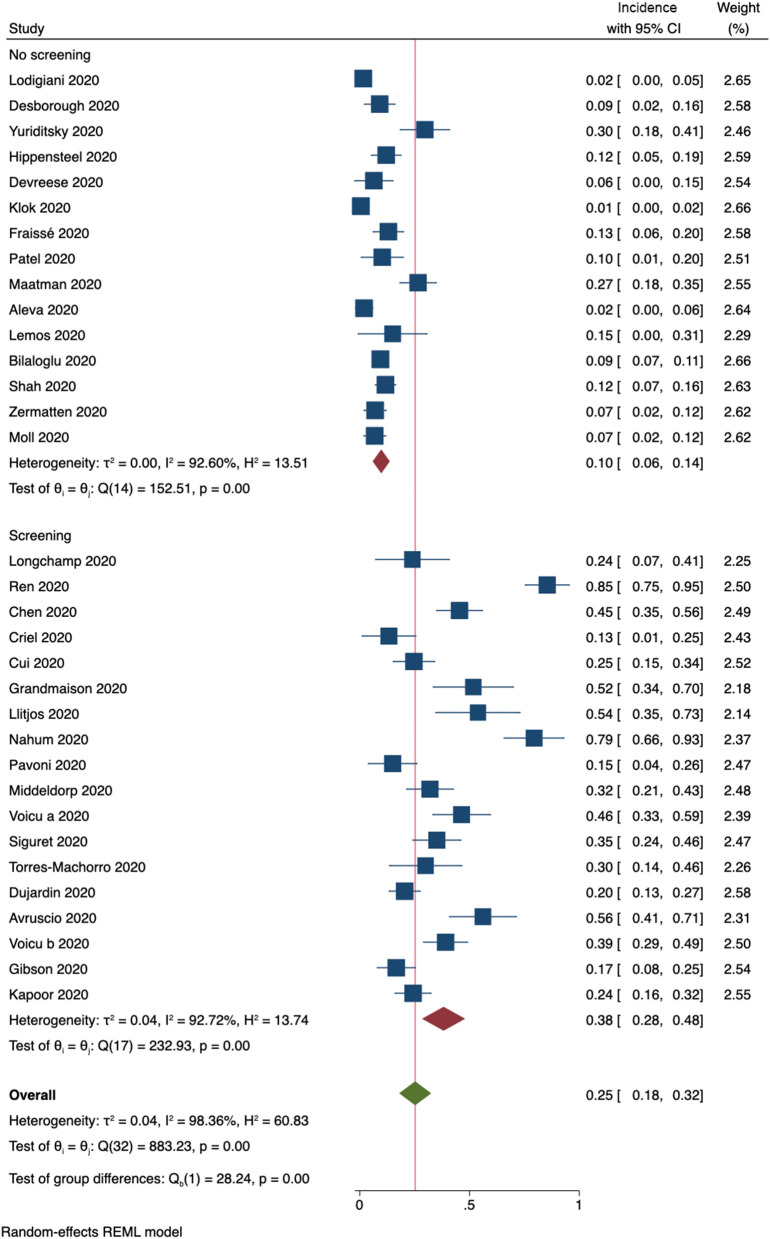
Incidence of any form of DVT (33 studies). Forest plot shows subgroups according to whether routine ultrasound screening for DVT was performed.

Thirty-three studies provided information on whether routine ultrasound screening for DVT was performed. Subgroup analysis resulted in a pooled incidence of 10% (95% CI, 6–14%, Figure 4) for 15 studies that did not perform screening. In contrast, studies that included ultrasound screening results were determined to have a pooled incidence of 38% (95% CI, 28–48%, Figure 4).

### Secondary Outcomes and Additional Subgroup Analyses

In total, 39 studies reported on the occurrence of ***any form of PE***, resulting in a pooled incidence of 12% (95% CI, 6–17%) for studies judged to have a moderate risk of bias, whereas the pooled incidence for studies with a high risk of bias was 13% (95% CI, 10% to 13%; Supplementary Figure 1). Heterogeneity was considerably higher (*I*^2^ = 90%) than for the outcome parameter of *clinically relevant PE*.

Sixteen studies reported the number of computer tomography (CT) scans performed to detect PE. In total, 514 CT scans were obtained for 1,433 patients. There was a strong positive correlation between the proportion of patients who underwent CT scans and the incidence of any form of PE (*R*^2^ = 69%, *p* < 0.001, Supplementary Figure 2A). In contrast, we did not find a correlation between the proportion of patients who underwent CT scans and the rate of non-subsegmental PE (*R*^2^ = 14%, *p* = 0.23, Supplementary Figure 2B).

Overall, the occurrence of ***any form of DVT***was reported in 40 studies. Studies judged to have a moderate risk of bias were determined to have a pooled incidence of 11% (95% CI, 6–16%), whereas studies with a high risk of bias were determined to have an incidence of 26% (95% CI, 18–34%, Supplementary Figure 3).

**Figure 5:**
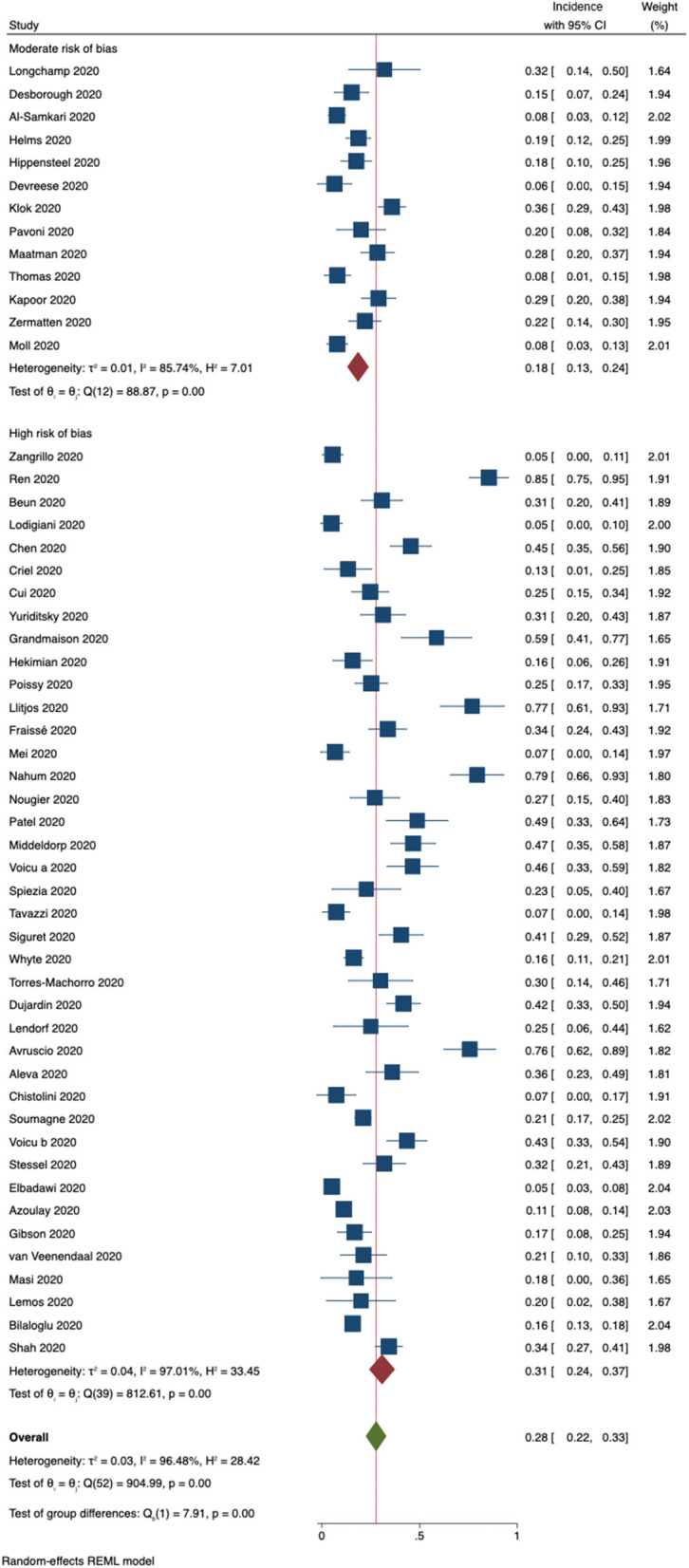
Incidence of the composite outcome VTE (DVT + PE) for 53 included studies. Forest plot shows subgroups according to risk of bias.

The pooled incidence of ***any form of VTE***, a composite outcome of any form of PE and DVT, was 18% (95% CI, 13–24%) in studies with a moderate risk of bias (Figure 5). In contrast, studies judged to have a high risk of bias were found to have a pooled VTE incidence of 31% (95% CI, 24–37%). One additional study explicitly reported not having observed VTE in the included patient cohort. Including this study in a sensitivity analysis using a mixed-effects model resulted in an overall incidence rate of 22% (95% CI, 16–28%).

Routine ultrasound screening was associated with an increase in the reported VTE incidence (Supplementary Figure 4), whereas larger study sample sizes showed a trend toward lower VTE incidence rates (Supplementary Figure 5). We did not observe differences in VTE incidence rates associated with the date of publication (Supplementary Figure 6). Supplementary Figure 7 shows no difference in pooled incidences of VTE for different subgroups according to anticoagulant regimen.

**Figure 6:**
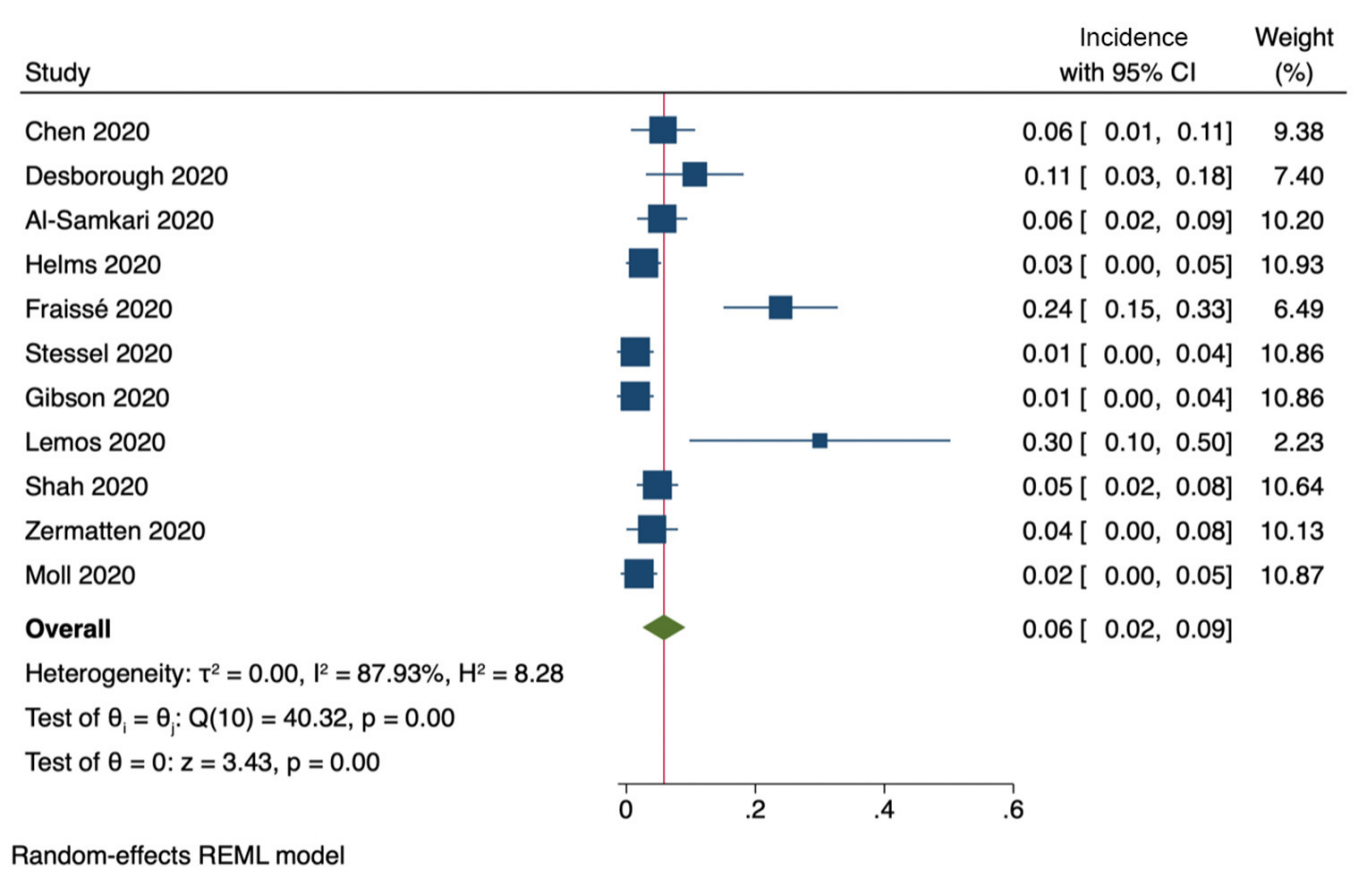
Incidence of clinically relevant bleeding (11 studies).

Eleven studies included information on the incidence of ***clinically relevant bleeding*** events (Figure 6). The pooled incidence was 6% (95% CI, 2–9%). Furthermore, six studies reported on the incidence of intracranial bleeding, with a pooled incidence of 2% (95% CI, 0.6–2.4%).

## Discussion

To the best of our knowledge, the current study is the first systematic review and meta-analysis to focus not only on the crude incidence but on the *clinical relevance* of VTE in critically ill patients with COVID-19. Current guidelines recognize risks associated with a potential overdiagnosis of incidental subsegmental PE and distal DVT that might be of questionable clinical relevance (74, 75). Therefore, we extracted data on the rates of clinically relevant PE and proximal DVT and examined the possible influence of different diagnostic approaches on the reported incidences. We found incidence estimates of 8, 14, and 18% for PE, DVT, and VTE, respectively. We could demonstrate the substantial influence of a high rate of CT scans and routine ultrasound screening on reporting higher incidences of isolated subsegmental PE and isolated distal DVT, respectively. Furthermore, we are the first to report a pooled incidence rate of 6% for clinically relevant bleeding and of 2% for intracranial bleeding in this specific patient cohort.

Overall, the included studies reported on a patient group with a collective high baseline risk of VTE. Patients were critically ill and thus bedridden, with reported mean ICU LOS considerably longer than 7 days. In studies that provided detailed patient characteristics, the majority of patients were at an advanced age and overweight. In addition to critical illness, immobility, advanced age and obesity have all previously been associated with increased VTE risk (76). VTE has thus long been recognized as a serious problem in critically ill patients (77). Hence, the VTE incidence reported in the current study needs to be viewed primarily in light of these relevant background factors and not only the COVID-19 disease. Additionally, infection *per se* is another risk factor for the occurrence of VTE. Severe COVID-19 disease is accompanied by excessive cytokine release, which in turn activates the coagulation cascade, resulting in typical laboratory alterations such as elevated fibrinogen and D-dimer levels (78). The close connection between inflammation and coagulation—immunothrombosis—has been known for more than a century (79). It thus seems reasonable to compare our findings with VTE rates in critically ill patients with sepsis. A recent study reported a VTE incidence rate of 37% in critically ill patients with sepsis, despite pharmacological thromboprophylaxis (80). Notably, routine ultrasound screening for DVT was used in this study. We found a comparable incidence rate of 44% for any form of VTE in studies that applied ultrasound screening for DVT. Another study reported a VTE incidence of 21% in a subgroup of critically ill patients with sepsis and acute respiratory distress syndrome, despite pharmacological thromboprophylaxis (81). In line with this, we found a pooled incidence of any form of VTE between 18 and 31%.

We observed a substantial degree of heterogeneity among the included studies. The reported VTE incidences in critically ill patients with COVID-19 vary widely, ranging from 0 to 85% (51, 52). A possible explanation and an important challenge when pooling the reported incidences is that different studies use distinct outcome definitions. For instance, only a subgroup of the studies reporting PE as an outcome parameter provided information to further characterize the form of PE. From a clinician's point of view, however, it is important to distinguish between a symptomatic patient with a central PE on one end of the spectrum and the incidental finding of a subsegmental PE in an asymptomatic patient on the other end of the spectrum. Along these lines, another possible explanation for the pronounced heterogeneity lies in the different diagnostic approaches. Current guidelines explicitly recommend against routine ultrasound screening for DVT in critically ill patients (10, 11). Interim guidance for the management of VTE in patients with COVID-19 adopted this recommendation (13). The underlying rationale for this recommendation is that routine screening might lead to the detection of asymptomatic, isolated distal DVT of questionable clinical relevance, which in turn might prompt the use of therapeutic anticoagulation in these patients and increase their bleeding risk. Nineteen of the 43 included studies that reported DVT incidence used ultrasound screening for the detection of DVT. Of note, both the DVT and overall VTE incidences were significantly higher in studies with screening than in studies without screening. Interestingly, when we pooled the incidences of proximal DVT, we found an incidence comparable with that in studies without ultrasound screening. A possible explanation may be the incidental detection of a high number of isolated distal DVT cases in screening studies. In line with this, a recent publication reported a 4-fold increase of isolated DVT with the use of ultrasound screening in COVID-19 patients in comparison with no screening (82). Similarly, the overall rate of any form of PE was positively correlated with the proportion of patients undergoing CT scans. Notably, this was not the case for the outcome parameter of non-subsegmental PE. Studies that provided details on the number of CT scans performed reported 514 CT scans in 1,433 patients. We hypothesize that the incidental detection of subsegmental PE of questionable clinical relevance in a substantial number of patients might have been caused by the high proportion of patients who underwent CT scans for other reasons. Recent publications highlight that the ideal diagnostic approach for the detection of VTE in patients with COVID-19 still remains unclear (17, 83).

Our work stands in contrast to previously published review articles that reported considerably higher incidence estimates for VTE in patients with COVID-19, particularly in those who are critically ill (84–91). For example, Shi et al. reported an estimated PE incidence of 19% for critically ill patients with COVID-19 (84). Similar incidence estimates (16–20%) were found by other meta-analyses as well (86, 87, 89). We report a significantly lower incidence estimate (8%) for clinically relevant PE. Similarly, we found a lower pooled incidence rate for proximal DVT (14%) than that determined in previous meta-analyses, which reported incidence rates of up to 33% for any form of DVT (87). On the one hand, these discrepancies can be explained by the use of different outcome definitions, as we specifically focused on the clinical relevance of VTE. On the other hand, we also found lower overall incidences for any form of PE (13%) and DVT (22%) than earlier meta-analyses. This might be explained by the larger number of included studies in our work, with the notable inclusion of more recent studies. In contrast to an earlier review article, we did not observe a trend toward a lower VTE incidence over time (92). However, we did find that reported VTE incidence rates decreased as the study sample size increased. Especially among the first published studies, most contained small sample sizes and the majority of data originated from centers overwhelmed with an unexpectedly high number of severely ill patients with COVID-19.

Another relevant aspect that distinguishes the current work from previously published review articles is that we calculated pooled incidence estimates for bleeding episodes. Critically ill patients carry an inherent bleeding risk that needs to be weighed against the thromboembolic risk when administering pharmacological thromboprophylaxis. Importantly, the pooled incidence of 6% for clinically relevant bleeding episodes was not much lower than the rate of clinically relevant PE (8%).

Regarding the high heterogeneity of anticoagulant regimens reported in the included studies, it is noteworthy that in a corresponding subgroup-analysis, we did not observe differences in VTE incidence. However, it needs to be stressed that the current meta-analysis was not intended to detect differences in efficacy between distinct anticoagulation strategies. To date, only one prospective, randomized, controlled trial has compared different anticoagulation regimens in critically ill patients (*n* = 20) with COVID-19 (36). Therefore, it seems unlikely that a meta-analysis could shed light on this important question at this point. In line with this, a recently published Cochrane review concluded that there is currently insufficient evidence to determine the risks and benefits of anticoagulation in patients with COVID-19 (15).

Despite having a number of strengths, such as the focus on clinically relevant VTE, including data from different centers around the world and the considerable number of included patients, relevant limitations of our work need to be recognized. First, we observed substantial heterogeneity among studies that—apart from distinct outcome definitions—may have been caused by differences in study designs and settings. In particular, the absence of uniform diagnostic procedures to detect VTE needs to be borne in mind when interpreting the results of our study. Furthermore, we cannot exclude that the different included patient cohorts and different treatment strategies used in studies might have resulted in distinct VTE risks. Second, the inherent limitations of retrospective data reporting applied to the majority of the included studies. This is a likely explanation for our finding that all of the included studies had a moderate to high risk of bias. Third, particularly with regard to the earliest studies publication bias and small-study effects might have influenced our results.

In conclusion, the present study summarizes the globally available evidence on the incidence of clinically relevant VTE and bleeding events in critically ill patients with COVID-19. We calculated the incidences of PE and DVT separately and found significantly lower incidence rates than previous meta-analyses when focusing on clinically relevant event rates. Reported incidence rates varied to a high degree according to different diagnostic approaches. Considerable knowledge gaps remain, particularly with regard to the influence of different anticoagulant dosing regimens on VTE incidence. Future research is urgently needed to address this question by applying high-quality research standards, including the application of uniform outcome definitions, to guarantee comparability between studies. Meanwhile, the results of our study provide clinically important information with respect to an individual risk-benefit assessment of anticoagulant use in critically ill patients with COVID-19.

## Data Availability Statement

The raw data supporting the conclusions of this article will be made available by the authors, without undue reservation.

## Author Contributions

JG was involved in the design of the study, literature review, data extraction, risk of bias assessment, statistical analysis, and drafted the manuscript. MW was involved in the design of the study, literature review, data extraction, and risk of bias assessment. MM was involved in the design of the study, data extraction, statistical analysis, and provided methodological support. HH was involved in the design of the study and the risk of bias assessment, provided methodological support, and undertook statistical analysis. HS was involved in the design of the study and literature review. EC was involved in the design of the study and performed the literature search. PK was involved in the design of the study and literature review. ES was involved in the design of the study, literature review, and data extraction. All authors contributed substantially to the writing of the manuscript, revised it, and approved it.

## Conflict of Interest

The authors declare that the research was conducted in the absence of any commercial or financial relationships that could be construed as a potential conflict of interest.

## Supplementary Material

Additional File 1 Search strategy. Additional file 1 shows the detailed literature search performed in MEDLINE via OVID.

Additional File 2 Supplementary Tables 1–3 show detailed study and patient characteristics and detailed quality assessments.

Additional File 3 Supplementary Figures 1–7 show additional subgroup analyses and correlations performed for secondary outcome parameters.
