# Supplementary material for: Risk of Clinically Relevant Venous Thromboembolism in Critically Ill Patients With COVID-19: A Systematic Review and Meta-Analysis
Source: Front Med (Lausanne). 2021 Mar 9;8:647917. doi: 10.3389/fmed.2021.647917 (PMC7985162; doi:10.3389/fmed.2021.647917)
Supplement: Additional File 1 — Search strategy. Additional file 1 shows the detailed literature search performed in MEDLINE via OVID. [file Data_Sheet_1.PDF]

Additional file 1: Search strategy

Literature search undertaken in MEDLINE via OVID on July 22, 2020. The same search strategy was used on October 7, 2020. Comparable search strategies were adopted for EMBASE, CENTRAL and Web of Science.

Database: Ovid MEDLINE(R) ALL <1946 to July 21, 2020>
Search Strategy:
----
1 exp coronavirus infections/ (22673)
2 exp Coronavirus/ (22444)
3 (coronavir* or corona vir*).ti,ot,ab,kf. (26352)
4 ((covid or ncov or n-cov or cov or coronavir* or corona vir* or betacoronavir*) adj3 ("19?" or "2019*" or wuhan or novel* or new or newly)).ti,ot,ab,kf. (34672)
5 (covid-19* or covid19* or covid-2019* or covid2019*).mp. [mp=title, abstract, original title, name of substance word, subject heading word, floating sub-heading word, keyword heading word, organism supplementary concept word, protocol supplementary concept word, rare disease supplementary concept word, unique identifier, synonyms] (33760)
6 (2019-ncov* or 2019-n-cov* or 2019ncov* or 2019n-cov* or 2019-novel-cov* or ncov2019* or n-cov2019* or ncov-2019* or n-cov-2019* or novel cov-2019* or novel cov-19* or ncov19* or n-cov19* or ncov-19* or n-cov-19*).mp. [mp=title, abstract, original title, name of substance word, subject heading word, floating sub-heading word, keyword heading word, organism supplementary concept word, protocol supplementary concept word, rare disease supplementary concept word, unique identifier, synonyms] (1018)
7 (sars-cov-2* or sars-cov2* or sars covid-2* or sarscov2* or sars-2-cov*).mp. [mp=title, abstract, original title, name of substance word, subject heading word, floating sub-heading word, keyword heading word, organism supplementary concept word, protocol supplementary concept word, rare disease supplementary concept word, unique identifier, synonyms] (10898)
8 (severe acute respiratory syndrome coronavirus 2* or severe acute respiratory syndrome cov* 2* or severe acute respiratory syndrome coronavirus 2019* or severe acute respiratory syndrome cov* 2019* or severe acute respiratory syndrome coronavirus 19* or severe acute respiratory syndrome cov* 19*).mp. [mp=title, abstract, original title, name of substance word, subject heading word, floating sub-heading word, keyword heading word, organism supplementary concept word, protocol supplementary concept word, rare disease supplementary concept word, unique identifier, synonyms] (13091)
9 wuhan virus*.ti,ot,ab,kf. (8)
10 hcov-19.mp. [mp=title, abstract, original title, name of substance word, subject heading word, floating sub-heading word, keyword heading word, organism supplementary concept word, protocol supplementary concept word, rare disease supplementary concept word, unique identifier, synonyms] (11)
11 (covid or ncov* or n-cov*).mp. [mp=title, abstract, original title, name of substance word, subject heading word, floating sub-heading word, keyword heading word, organism supplementary concept word, protocol supplementary concept word, rare disease supplementary concept word, unique identifier, synonyms] (34100)
12 1 or 2 or 3 or 4 or 5 or 6 or 7 or 8 or 9 or 10 or 11 (55483)
13 exp "embolism and thrombosis"/ (218751)
14 ((pulmonary or lung*) adj3 (embol* or thromboemboli* or thrombo emboli*)).ti,ot,ab,kf. (48146)
15 ((lung* or pulmonary) adj3 (clot* or thrombus or thrombi or microthromb*)).ti,ot,ab,kf. (1420)
16 pulmonary infarct*.ti,ot,ab,kf. (1046)
17 pe.ti,ot,ab,kf. and (pulmon* or lung*).mp. [mp=title, abstract, original title, name of substance word, subject heading word, floating sub-heading word, keyword heading word, organism supplementary concept word, protocol supplementary concept word, rare disease supplementary concept word, unique identifier, synonyms] (10136)
18 embol*.ti,ot,ab,kf. (135676)
19 (thromboemboli* or thromb* emboli*).ti,ot,ab,kf. (64863)
20 (thrombos#s* or thrombosed or thrombotic* or thrombolic*).ti,ot,ab,kf. (170278)
21 (dvt or vte).ti,ot,ab,kf. (19528)
22 ((venous* or veno?s* or vein*) adj1 (thrombos#s* or thrombotic* or thromboemboli* or thrombo emboli* or thrombus or thrombi or microthromb*)).ti,ot,ab,kf. (68363)
23 (blood adj2 clot*).ti,ot,ab,kf. (10538)
24 exp Hemostasis/ (113307)
25 exp Blood Coagulation Disorders/ (96374)
26 exp Anticoagulants/ (220982)
27 (h?emostasis* or blood stasis* or h?emostatic*).ti,ot,ab,kf. (53732)
28 (coagulopath* or coagula* or hypercoagula*).ti,ot,ab,kf. (139970)
29 ((intravascular* or intra vascular* or intravasal* or intra vasal* or diffuse*) adj3 (clot* or coagula* or hypercoagula* or agglutina*)).ti,ot,ab,kf. (12390)
30 dic.ti,ot,ab,kf. (8460)
31 (anticoagula* or anti coagula* or antithrombotic* or anti thrombotic* or thromboprophyla* or thromb* prophyla* or thromboly*).ti,ot,ab,kf. (149819)
32 ((prevent* or prophyl*) adj3 (thromb* or embol* or pe or vte or dvt)).ti,ot,ab,kf. (23285)
33 heparin/ (54220)
34 exp heparin, low molecular weight/ (12497)

35 heparin?.ti,ot,ab,kf,nm,rn. (98470)
36 (weight adj6 heparin?).ti,ot,ab,kf. (13840)
37 (lmwh or lmwhs or low molecular weight heparin?).ti,ot,ab,kf,nm,rn. (13152)
38 (nadroparin or fraxiparin* or fraxodi or seleparin*).ti,ot,ab,kf,hw,nm,rn. (809)
39 (ufh or ufhs or unfractionated heparin?).ti,ot,ab,kf,nm,rn. (6179)
40 (doac or doacs or (direct acting adj3 (anticoagulant* or anti coagulant*))).ti,ot,ab,kf,nm,rn. (2289)
41 (fondaparinux or arixtra or fondaparin or quixidar).ti,ot,ab,kf,hw,nm,rn. (1974)
42 or/13-41 (852464)
43 12 and 42 (1476)
44 exp Intensive Care Units/ (84417)
45 exp Critical Care/ (57782)
46 Critical Illness/ (28923)
47 (icu? or (intensiv* adj3 care)).mp. [mp=title, abstract, original title, name of substance word, subject heading word, floating sub-heading word, keyword heading word, organism supplementary concept word, protocol supplementary concept word, rare disease supplementary concept word, unique identifier, synonyms] (193405)
48 (respiratory adj3 care unit?).mp. [mp=title, abstract, original title, name of substance word, subject heading word, floating sub-heading word, keyword heading word, organism supplementary concept word, protocol supplementary concept word, rare disease supplementary concept word, unique identifier, synonyms] (1528)
49 (critical* adj3 (ill* or care or unit?)).ti,ot,ab,kf,jw. (161333)
50 care unit?.mp. [mp=title, abstract, original title, name of substance word, subject heading word, floating sub-heading word, keyword heading word, organism supplementary concept word, protocol supplementary concept word, rare disease supplementary concept word, unique identifier, synonyms] (167999)
51 ((intensiv* adj3 care) or icu?).jw. (24341)
52 Severe Acute Respiratory Syndrome/ (4756)
53 exp Respiration, Artificial/ (76858)
54 exp Extracorporeal Circulation/ (83027)
55 Extracorporeal Membrane Oxygenation/ (10506)
56 exp Shock/ (74763)
57 Respiratory Distress Syndrome, Adult/ (19485)
58 ((artificial* or mechanical* or invasiv* or noninvasiv* or non-invasiv* or high-frequency or positive pressure or positive airway or positive end expiratory pressure) adj3 (respiration* or ventilat*)).ti,ot,ab,kf. (72698)
59 (positive adj4 (pressure or support) adj4 (ventilation* or respiration*)).ti,ot,ab,kf. (8302)
60 (nippv or nppv or ippv).ti,ot,ab,kf. (1790)
61 niv.ti,ot,ab,kf. (3125)
62 (cpap or bipap or positive* airway pressure*).ti,ot,ab,kf. (14028)
63 (peep or positive end expiratory pressure).ti,ot,ab,kf. (8050)
64 (extracorpor?al adj3 circulation*).ti,ot,ab,kf. (8235)
65 (extracorpor?al adj3 oxygenation*).ti,ot,ab,kf. (11314)
66 (extracorpor?al adj3 life support?).ti,ot,ab,kf. (2185)
67 (ecmo or ecls).ti,ot,ab,kf. (8880)
68 ((circulatory or cardiovascular*) adj3 collapse*).ti,ot,ab,kf. (2901)
69 (shock or multi* organ failure*).ti,ot,ab,kf. (191060)
70 (respiratory distress syndrome adj4 (acute* or adult)).ti,ot,ab,kf. (18325)
71 (ards or ardss).ti,ot,ab,kf. (12624)
72 exp Emergency Medicine/ (13773)
73 Emergency Service, Hospital/ (68726)
74 (emergency adj (room? or department? or unit? or hospital? or ward? or medicine)).ti,ot,ab,kf. (125737)
75 emergency medicine.jw. (63802)
76 or/44-75 (873690)
77 43 and 76 (597)
78 (coronavir* or corona vir*).ti,ot. (12339)
79 ((covid or ncov or n-cov or cov or coronavir* or corona vir* or betacoronavir*) adj3 ("19?" or "2019*" or wuhan or novel* or new or newly)).ti,ot. (29805)
80 (covid-19* or covid19* or covid-2019* or covid2019*).ti,ot. (27220)
81 (2019-ncov* or 2019-n-cov* or 2019ncov* or 2019n-cov* or 2019-novel-cov* or ncov2019* or n-cov2019* or ncov-2019* or n-cov-2019* or novel cov-2019* or novel cov-19* or ncov19* or n-cov19* or ncov-19* or n-cov-19*).ti,ot. (339)
82 (sars-cov-2* or sars-cov2* or sars covid-2* or sarscov2* or sars-2-cov*).ti,ot. (4679)
83 (severe acute respiratory syndrome coronavirus 2* or severe acute respiratory syndrome cov* 2* or severe acute respiratory syndrome coronavirus 2019* or severe acute respiratory syndrome cov* 2019* or severe acute respiratory syndrome coronavirus 19* or severe acute respiratory syndrome cov* 19*).ti,ot. (315)
84 wuhan virus*.ti,ot. (3)
85 (covid or ncov* or n-cov*).ti,ot. (27655)
86 or/78-85 (41499)
87 ((pulmonary or lung*) adj3 (embol* or thromboemboli* or thrombo emboli*)).ti,ot. (22420)
88 ((lung* or pulmonary) adj3 (clot* or thrombus or thrombi or microthromb*)).ti,ot. (369)
89 pulmonary infarct*.ti,ot. (485)

90 pe.ti,ot. (2101)
91 embol*.ti,ot. (57692)
92 (thromboemboli* or thromb* emboli*).ti,ot. (23079)
93 (thrombos#s* or thrombosed or thrombotic* or thrombolic*).ti,ot. (61248)
94 (dvt or vte).ti,ot. (1243)
95 ((venous* or veno?s* or vein*) adj1 (thrombos#s* or thrombotic* or thromboemboli* or thrombo emboli* or thrombus or thrombi or microthromb*)).ti,ot. (33099)
96 (blood adj2 clot*).ti,ot. (2027)
97 (h?emostasis* or blood stasis* or h?emostatic*).ti,ot. (15764)
98 (coagulopath* or coagula* or hypercoagula*).ti,ot. (42286)
99 ((intravascular* or intravasal* or diffuse*) adj3 (clot* or coagula* or hypercoagula* or agglutina*)).ti,ot. (4654)
100 dic.ti,ot. (1085)
101 (anticoagula* or anti coagula* or antithrombotic* or anti thrombotic* or thromboprophyla* or thromb* prophyla* or thromboly*).ti,ot. (56302)
102 ((prevent* or prophyl*) adj3 (thromb* or embol* or pe or vte or dvt)).ti,ot. (7416)
103 (heparin? or lmwh or lmwhs or ufh or ufhs).ti,ot. (31437)
104 (doac or doacs or (direct acting adj3 (anticoagulant* or anti coagulant*))).ti,ot. (303)
105 (nadroparin or fraxiparin* or fraxodi or seleparin*).ti,ot. (209)
106 (fondaparinux or arixtra or fondaparin or quixidar).ti,ot. (601)
107 or/87-106 (269535)
108 86 and 107 (645)
109 77 or 108 (1031)
110 limit 109 to yr="2020" (973)
111 limit 110 to english (938)
112 limit 111 to (case reports or editorial) (162)
113 limit 112 to (adaptive clinical trial or classical article or clinical conference or clinical study or clinical trial, all or clinical trial, phase i or clinical trial, phase ii or clinical trial, phase iii or clinical trial, phase iv or clinical trial protocol or clinical trial protocols as topic or clinical trial or comment or comparative study or congress or controlled clinical trial or "corrected and republished article" or dataset or equivalence trial or evaluation study or "expression of concern" or government publication or introductory journal article or journal article or letter or multicenter study or observational study or overall or pragmatic clinical trial or published erratum or randomized controlled trial or "research support, american recovery and reinvestment act" or research support, nih, extramural or research support, nih, intramural or research support, non us gov't or research support, us gov't, non phs or research support, us gov't, phs or technical report or twin study or validation study) (60)
114 112 not 113 (102)
115 111 not 114 (836)
116 exp animals/ not exp humans/ (4719652)
117 115 not 116 (835)

****
