# Supplementary material for: Risk of Clinically Relevant Venous Thromboembolism in Critically Ill Patients With COVID-19: A Systematic Review and Meta-Analysis
Source: Front Med (Lausanne). 2021 Mar 9;8:647917. doi: 10.3389/fmed.2021.647917 (PMC7985162; doi:10.3389/fmed.2021.647917)
Supplement: Additional File 2 — Supplementary Tables 1–3 show detailed study and patient characteristics and detailed quality assessments. [file Data_Sheet_2.PDF]

Additional file 2

Supplementary Table S1: Study characteristics

Table S1 shows detailed characteristics of the 54 included studies. IRB: institutional review board; NR: not reported.

First author	Month of publication	Country	Study design	IRB approval	No of patients
**Al-Samkari**	July 2020	USA	retrospective, multicenter	yes	144
**Aleva**	August 2020	Netherlands	retrospective; single-center	inf. consent waived	50
**Avruscio**	Septemb 2020	Italy	prospective; observational; single-center	yes	41
**Azoulay**	August 2020	France	retrospective; multicenter	inf. consent waived	376
**Beun**	April 2020	Netherlands	retrospective, single-center	inf. consent waived	75
**Bilaloglu**	August 2020	USA	retrospective; multicenter	inf. consent waived	829
**Chen**	June 2020	China	retrospective, single-center	inf. consent waived	88
**Chistolini**	July 2020	Italy	prospective; observational; single-center	yes	27
**Criel**	July 2020	Belgium	prospective, observational, single-center	NR	30
**Cui**	May 2020	China	retrospective, single-center	yes	81
**Desborough**	May 2020	UK	retrospective, single-center	yes	66
**Devreese**	July 2020	Belgium	prospective, observational, single-center	yes	31
**Dujardin**	September 2020	Netherlands	retrospective; single-center	no	127
**Elbadawi**	September 2020	USA	retrospective; multicenter	yes	296
**Fraissé**	June 2020	France	retrospective, single-center	yes	92
**Gibson**	September 2020	USA	prospective; observational; single-center	NR	72
**Grandmaison**	June 2020	Switzerland	prospective, observational, single-center	NR	29
**Hekimian**	June 2020	France	retrospective, single-center	informed consent	51
**Helms**	May 2020	France	prospective, observational, multicenter, control	yes	150
**Hippensteel**	June 2020	USA	retrospective, single-center	yes	91
**Kapoor**	October 2020	USA	prospective; observational; multicenter	yes	107
**Klok**	April 2020	Netherlands	retrospective, multicenter	inf. consent waived	184
**Lemos**	September 2020	Brazil	prospective; interventional; single-center	informed consent	20
**Lendorf**	August 2020	Denmark	retrospective; single-center	inf. consent waived	20
**Llitjos**	May 2020	France	retrospective, multicenter	yes	26
**Lodigiani**	April 2020	Italy	retrospective, single-center	informed consent	61
**Longchamp**	May 2020	Switzerland	prospective, observational, single-center	inf. consent waived	25
**Maatman**	May 2020	USA	retrospective, multicenter	inf. consent waived	109
**Masi**	June 2020	France	prospective; observational; single-center;control	informed consent	17
**Mei**	July 2020	China	retrospective, single-center, control	inf. consent waived	45
**Middeldorp**	May 2020	Netherlands	retrospective, single-center	inf. consent waived	75
**Moll**	July 2020	USA	retrospective; single-center	yes	102
**Nahum**	May 2020	France	prospective, observational, single-center	inf. consent waived	34
**Nougier**	July 2020	France	prospective, observational, single-center	informed consent	48
**Patel**	July 2020	UK	retrospective, single-center	inf. consent waived	39
**Pavoni**	May 2020	Italy	retrospective, single-center	inf. consent waived	40
**Poissy**	April 2020	France	retrospective, single-center, control	yes	107
**Ren**	July 2020	China	prospective, observational, multicenter	informed consent	48
**Ranucci**	May 2020	Italy	prospective, observational, single-center	yes	16
**Shah**	September 2020	UK	retrospective; multicenter	no	187
**Siguret**	July 2020	France	prospective, observational, single-center	yes	74
**Soumagne**	June 2020	France/Belgium	retrospective; multicenter	inf. consent waived	375
**Spiezia**	April 2020	Italy	observational, single-center	informed consent	22
**Stessel**	July 2020	Belgium	prospective; interventional; single-center	inf. consent waived	72
**Tavazzi**	April 2020	Italy	observational, single-center	informed consent	54
**Thomas**	April 2020	UK	retrospective, single-center	inf. consent waived	63
**Torres-Machorro**	September 2020	Mexico	prospective; observational; single-center	informed consent	30
**van Veenendaal**	September 2020	Netherlands	retrospective; single-center	NR	47
**Voicu (a)**	May 2020	France	prospective, observational, single-center	informed consent	56
**Voicu (b)**	September 2020	France	prospective; observational; single-center	yes	92
**Whyte**	July 2020	UK	retrospective, single-center	inf. consent waived	222
**Yuriditsky**	June 2020	USA	retrospective, single-center	inf. consent waived	64
**Zangrillo**	April 2020	Italy	retrospective, single-center	yes	73
**Zermatten**	August 2020	Switzerland	retrospective; single-center	informed consent	100

Supplementary Table S2: Risk of bias assessment

Table S2 depicts the detailed quality assessment for the 54 included studies.

First author	Question 1	Question 2	Question 3	Question 4	Question 5	Question 6a (PE)	Question 6b (DVT)	Question 7a (PE)	Question 7b (DVT)	Question 8a (PE)	Question 8b (DVT)	Question 9	Question 10	Question 11
Al-Samkari	1	1	1	1	1	1	1	1	1	0	0	0	1	moderate
Aleva	1	0	1	1	1	0	0	0	0	0	0	1	1	high
Avruscio	1	1	0	1	1	1	1	1	1	0	1	1	0	high
Azoulay	1	1	1	1	1	0	NR	0	NR	0	NR	0	0	high
Beun	1	0	1	1	1	0	0	1	1	0	0	0	1	high
Bilaloglu	1	1	1	1	0	0	0	0	0	0	0	0	1	high
Chen	1	0	0	1	1	NR	0	NR	1	NR	1	1	1	high
Chistolini	1	1	1	1	1	0	NR	0	NR	0	NR	0	0	high
Criel	1	1	1	1	1	NR	0	NR	1	NR	1	0	1	high
Cui	1	1	0	1	1	NR	0	NR	1	NR	0	0	1	high
Desborough	1	1	1	1	1	1	1	1	1	1	1	0	1	moderate
Devreese	1	1	1	1	1	NR	0	NR	1	NR	0	1	1	moderate
Dujardin	1	1	1	0	1	1	0	1	1	1	1	0	1	high
Elbadawi	1	1	1	1	1	NR	NR	NR	NR	NR	NR	0	1	high
Fraissé	1	1	1	1	1	0	0	0	0	0	0	0	1	high
Gibson	1	1	0	0	1	NR	0	NR	1	NR	1	0	1	high
Grandmaison	1	1	1	1	1	0	0	1	1	1	1	0	1	high
Hekimian	1	0	0	0	1	1	NR	1	NR	0	NR	0	0	high
Helms	1	1	0	1	1	1	0	1	0	1	0	0	1	moderate
Hippensteel	1	1	0	1	1	0	0	1	1	0	0	0	1	moderate
Kapoor	1	1	0	0	1	1	0	1	1	1	1	1	1	moderate
Klok	1	1	1	1	1	1	1	1	1	0	0	1	1	moderate
Lemos	1	1	0	0	1	0	0	0	0	0	0	0	1	high
Lendorf	1	1	1	1	1	0	NR	1	NR	0	NR	0	1	high
Llitjos	1	1	1	1	1	0	0	1	1	1	1	0	1	high
Lodigiani	1	1	1	0	1	0	1	1	1	1	1	0	0	high
Longchamp	1	0	1	1	1	1	1	1	1	1	1	0	1	moderate
Maatman	1	1	1	1	1	0	1	1	1	1	1	1	1	moderate
Masi	1	1	1	0	1	0	NR	0	NR	0	NR	0	0	high
Mei	1	1	1	1	1	0	0	0	0	1	1	0	1	high
Middeldorp	1	1	1	1	1	1	0	1	0	0	0	1	1	high
Moll	1	1	1	1	1	1	1	1	1	1	1	1	0	moderate
Nahum	1	1	1	1	1	NR	0	NR	1	NR	1	0	1	high
Nougier	1	1	0	0	1	0	0	0	0	0	0	0	1	high
Patel	1	0	0	1	1	0	0	1	1	1	0	0	1	high
Pavoni	1	1	1	1	1	0	0	1	1	0	1	0	1	moderate
Poissy	1	1	1	1	1	1	0	1	0	1	0	1	1	high
Ren	1	1	1	0	1	NR	1	NR	1	NR	1	1	1	high
Ranucci	1	0	0	1	1	0	0	0	0	0	0	0	0	high
Shah	1	1	1	1	1	0	0	1	1	1	1	1	1	high
Siguret	1	1	1	1	1	0	0	1	1	1	1	0	1	high
Soumagne	1	1	0	1	1	0	0	0	0	0	0	0	1	high
Spiezia	1	1	0	0	1	NR	0	NR	0	NR	0	0	1	high
Stessel	1	1	1	1	1	NR	0	NR	1	NR	1	0	0	high
Tavazzi	1	1	1	0	1	0	0	1	1	0	0	0	1	high
Thomas	1	1	1	1	1	1	1	1	1	1	1	0	1	moderate
Torres-Machorro	1	1	0	0	1	NR	0	NR	1	NR	1	0	1	high
Veenendal	1	1	0	0	1	1	1	0	0	1	1	1	1	high
Voicu (a)	1	1	0	0	1	NR	0	NR	1	NR	1	0	1	high
Voicu (b)	1	1	0	1	1	0	1	1	1	0	1	0	0	high
Whyte	1	0	0	1	1	1	NR	1	NR	1	NR	0	1	high
Yuriditsky	1	0	0	1	1	0	0	1	1	1	1	1	1	high
Zangrillo	1	0	1	1	1	0	NR	0	NR	0	NR	1	0	high
Zermatten	1	1	1	0	1	1	1	1	1	1	1	0	1	moderate

Supplementary Table S3: Patient characteristics

Table S3 shows relevant patient characteristics and type of pharmacological thromboprophylaxis/anticoagulation used. Data are presented as proportion or mean. BMI: body mass index; ICU LOS: intensive care unit length of stay; NR: not reported. Disease duration: duration of disease until ICU admission, *three studies reported the duration of disease until first venous thromboembolic event.

First author	Female %	Age years	BMI kg/m^2	Mortality %	ICU-LOS days	Disease duration days	Malignancy %	Anticoagulation
**Al-Samkari**	6	65	NR	19	NR	NR	NR	any dose heparin
**Aleva**	34	65	29	26	17	NR	4	prophylactic heparin
**Avruscio**	20	67	28	NR	27	NR	12	other
**Azoulay**	23	62	28	26	10	8	NR	NR
**Beun**	NR	NR	NR	NR	NR	NR	NR	high-dose heparin
**Bilaloglu**	NR	NR	NR	54	NR	NR	NR	prophylactic heparin
**Chen**	39	63	NR	23	22	28*	6	prophylactic heparin
**Chistolini**	37	66	NR	NR	NR	NR	NR	any dose heparin
**Criel**	33	65	30	13	NR	NR	NR	any dose heparin
**Cui**	54	60	NR	10	NR	NR	NR	none
**Desborough**	27	59	28	30	9	NR	8	any dose heparin
**Devreese**	10	63	NR	13	25	NR	19	any dose heparin
**Dujardin**	23	62	27	NR	NR	NR	NR	any dose heparin
**Elbadawi**	NR	NR	NR	NR	NR	NR	NR	NR
**Fraissé**	21	61	30	41	NR	17*	NR	any dose heparin
**Gibson**	21	64	NR	24	NR	NR	NR	any dose heparin
**Grandmaison**	38	66	28	NR	NR	NR	7	prophylactic heparin
**Hekimian**	NR	NR	NR	NR	NR	NR	NR	NR
**Helms**	19	63	NR	9	10	NR	6	any dose heparin
**Hippensteel**	42	57	33	24	NR	NR	3	any dose heparin
**Kapoor**	42	60	30	47	7	NR	10	other
**Klok**	24	64	NR	22	NR	NR	3	any dose heparin
**Lemos**	20	57	34	20	NR	NR	0	any dose heparin
**Lendorf**	85	64	29	25	NR	12	10	any dose heparin
**Llitjos**	23	68	30	12	NR	7	0	any dose heparin
**Lodigiani**	20	61	NR	NR	12	NR	3	prophylactic heparin
**Longchamp**	36	68	28	20	NR	10	8	prophylactic heparin
**Maatman**	43	61	35	25	13	8	NR	prophylactic heparin
**Masi**	29	48	31	NR	NR	NR	NR	prophylactic heparin
**Mei**	NR	NR	NR	NR	NR	NR	NR	prophylactic heparin
**Middeldorp**	23	62	27	NR	15	NR	4	any dose heparin
**Moll**	42	65	30	27	15	NR	21	prophylactic heparin
**Nahum**	26	62	31	NR	NR	NR	3	prophylactic heparin
**Nougier**	NR	63	29	NR	NR	NR	NR	prophylactic heparin
**Patel**	18	53	31	NR	NR	NR	NR	any dose heparin
**Pavoni**	40	61	28	13	8	12	NR	prophylactic heparin
**Poissy**	NR	NR	NR	14	NR	NR	NR	prophylactic heparin
**Ren**	46	70	NR	31	NR	NR	NR	prophylactic heparin
**Ranucci**	6	61	26	44	NR	NR	NR	other
**Shah**	34	57	28	32	15	NR	8	any dose heparin
**Siguret**	NR	64	29	26	NR	10	NR	any dose heparin
**Soumagne**	23	64	30	36	NR	NR	12	other
**Spiezia**	9	67	30	NR	NR	NR	0	prophylactic heparin
**Stessel**	32	67	26	26	12	NR	NR	any dose heparin
**Tavazzi**	NR	NR	NR	NR	NR	NR	NR	prophylactic heparin
**Thomas**	30	NR	NR	16	8	NR	2	prophylactic heparin
**Torres-Machorro**	23	62	28	NR	NR	NR	NR	any dose heparin
**van Veenendaal**	23	63	29	15	18	NR	NR	prophylactic heparin
**Voicu (a)**	25	NR	NR	NR	NR	NR	NR	prophylactic heparin
**Voicu (b)**	28	62	28	59	NR	12*	0	prophylactic heparin
**Whyte**	NR	NR	NR	NR	NR	NR	NR	any dose heparin
**Yuriditsky**	28	64	NR	30	NR	NR	0	any dose heparin
**Zangrillo**	16	61	27	23	11	9	3	other
**Zermatten**	26	64	NR	28	11	NR	3	any dose heparin
