# Supplementary material for: Risk of Clinically Relevant Venous Thromboembolism in Critically Ill Patients With COVID-19: A Systematic Review and Meta-Analysis
Source: Front Med (Lausanne). 2021 Mar 9;8:647917. doi: 10.3389/fmed.2021.647917 (PMC7985162; doi:10.3389/fmed.2021.647917)

Additional file 3

Supplementary figure S1: Incidences of any form of PE according to risk of bias

Fig. S1 shows the pooled incidence of any form of PE (39 studies) in comparison between subgroups according to risk of bias. One additional study explicitly reported not having observed PE in the included patients. Including this study in a sensitivity analysis using a mixed-effects model resulted in a pooled incidence of 12% (95% CI, 9% to 14%) for any form of PE.

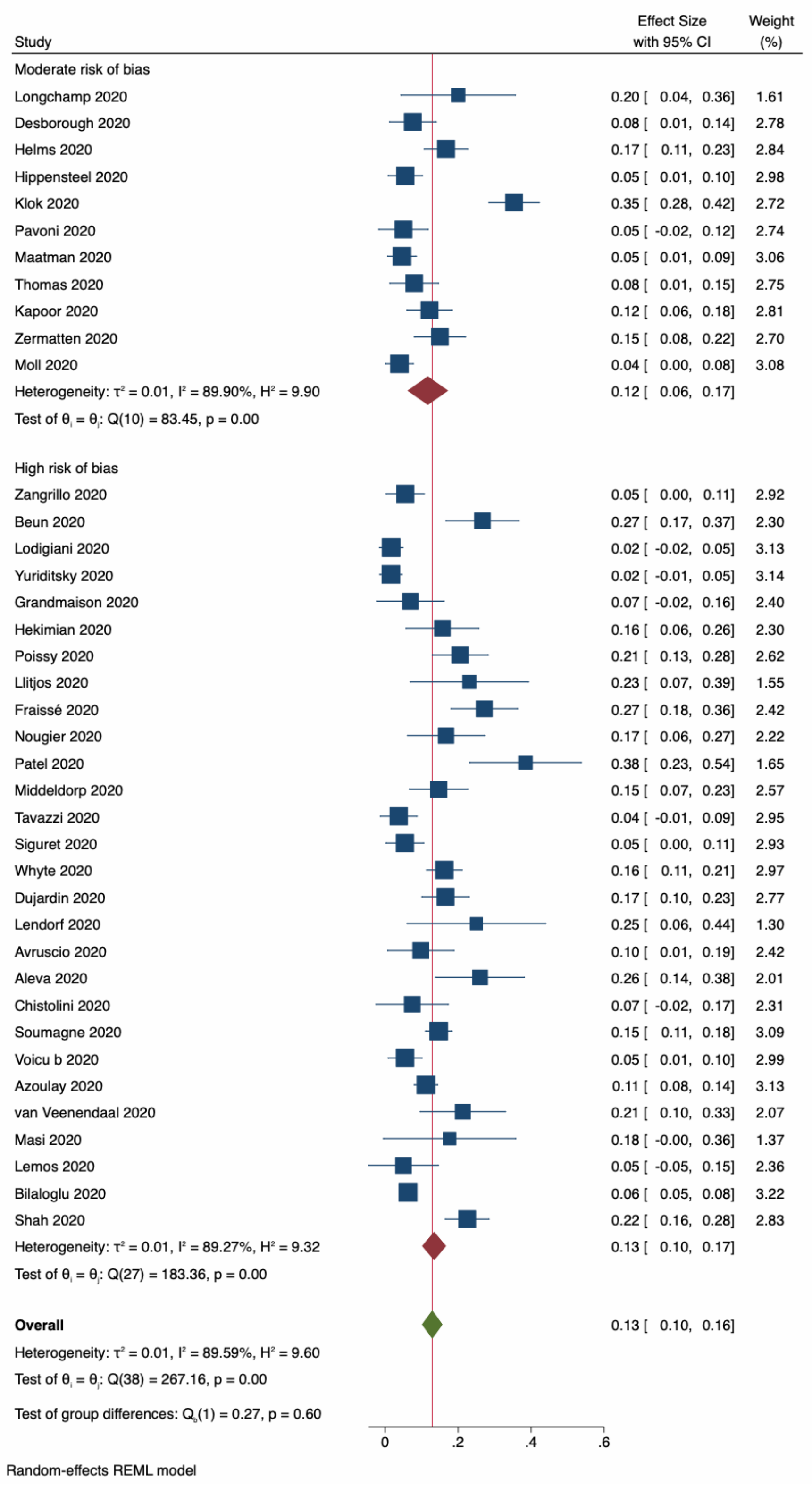

Supplementary figure S2: Correlation between proportion of CT scans and rate of PE

Fig. S2 shows the correlation between proportion of patients undergoing CT scans and (A) any form of PE, as well as (B) non-subsegmental PE.

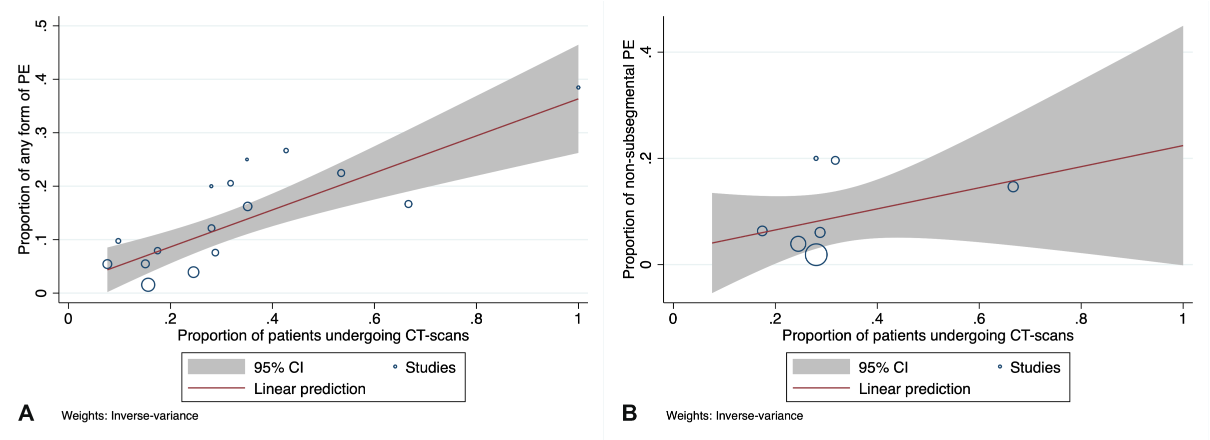

Supplementary figure S3: Incidences of any form of DVT according to risk of bias

Fig. S3 shows the pooled incidence of any form of DVT (40 studies) in comparison between subgroups according to risk of bias. Three additional studies explicitly reported not having observed DVT in the included patient cohort. Including these studies in a sensitivity analysis using a mixed-effects model resulted in a pooled incidence of 13% (95% CI, 9% to 18%).

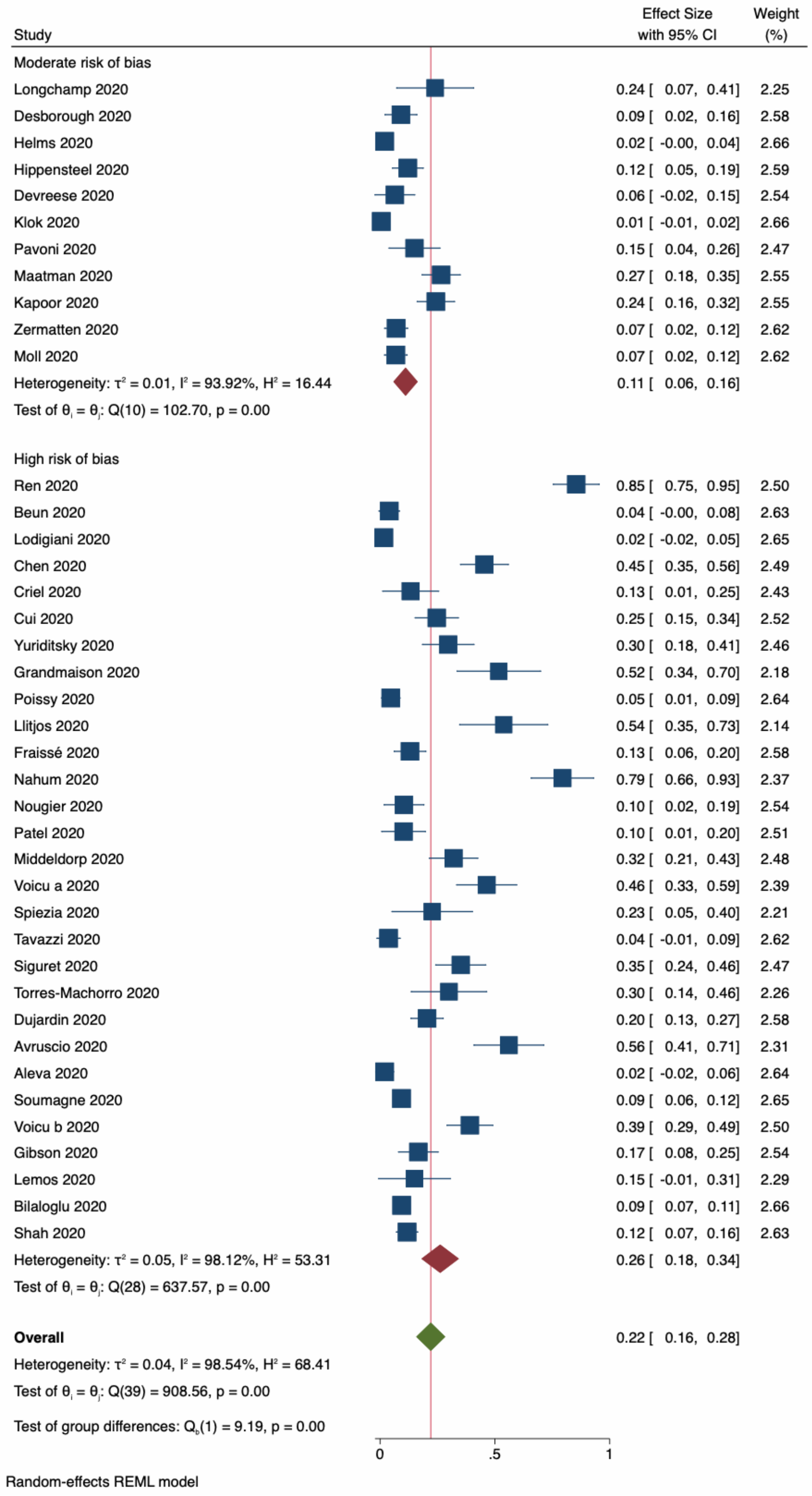

Supplementary figure S4: VTE incidence screening vs. no-screening

Fig. S4 shows the pooled incidence of VTE in comparison between subgroups with and without DVT ultrasound screening.

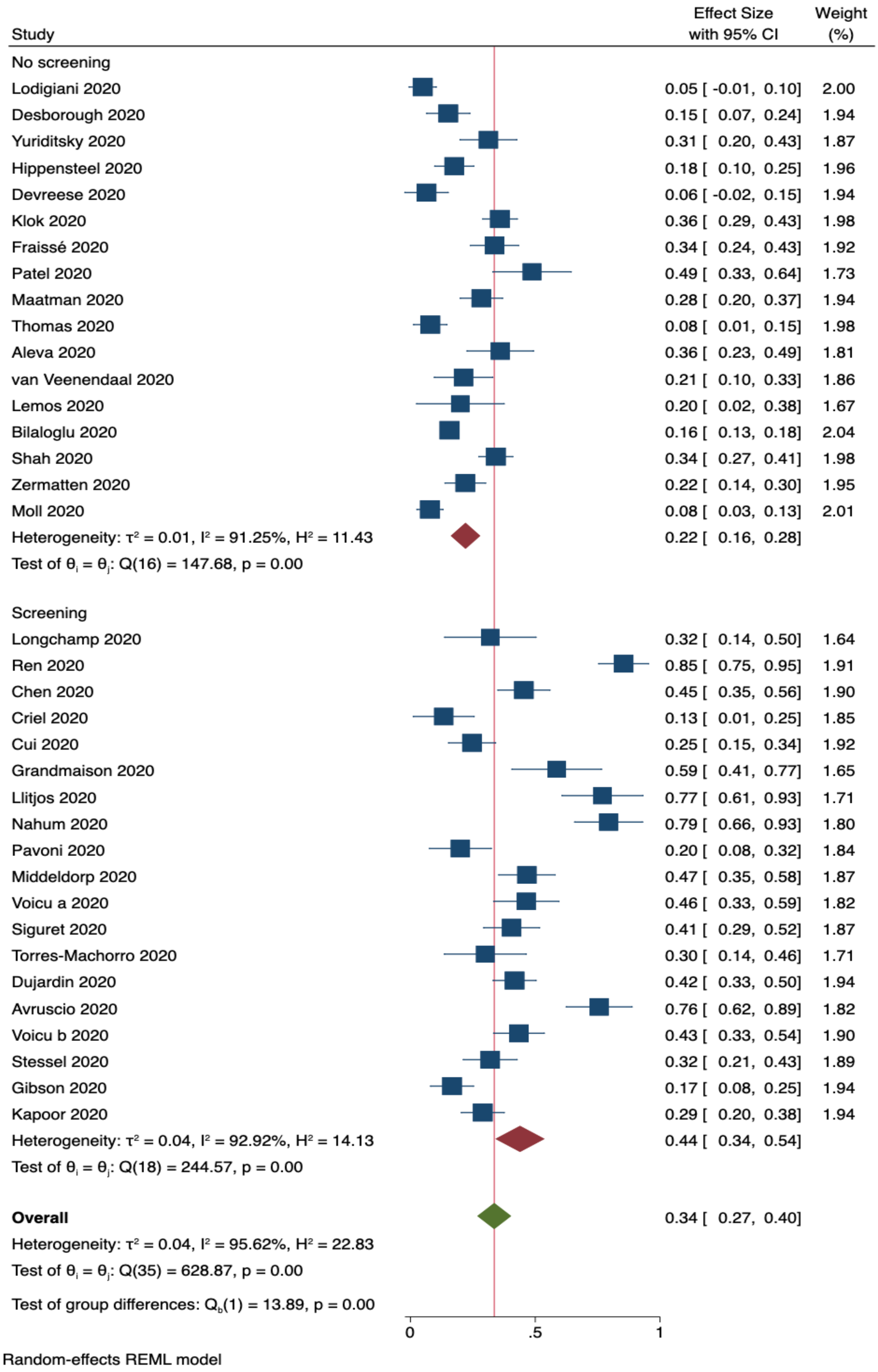

Supplementary figure S5: Correlation between study sample size and VTE incidence

Fig. S5 shows a trend towards decreasing reported VTE incidence with increasing study sample size.

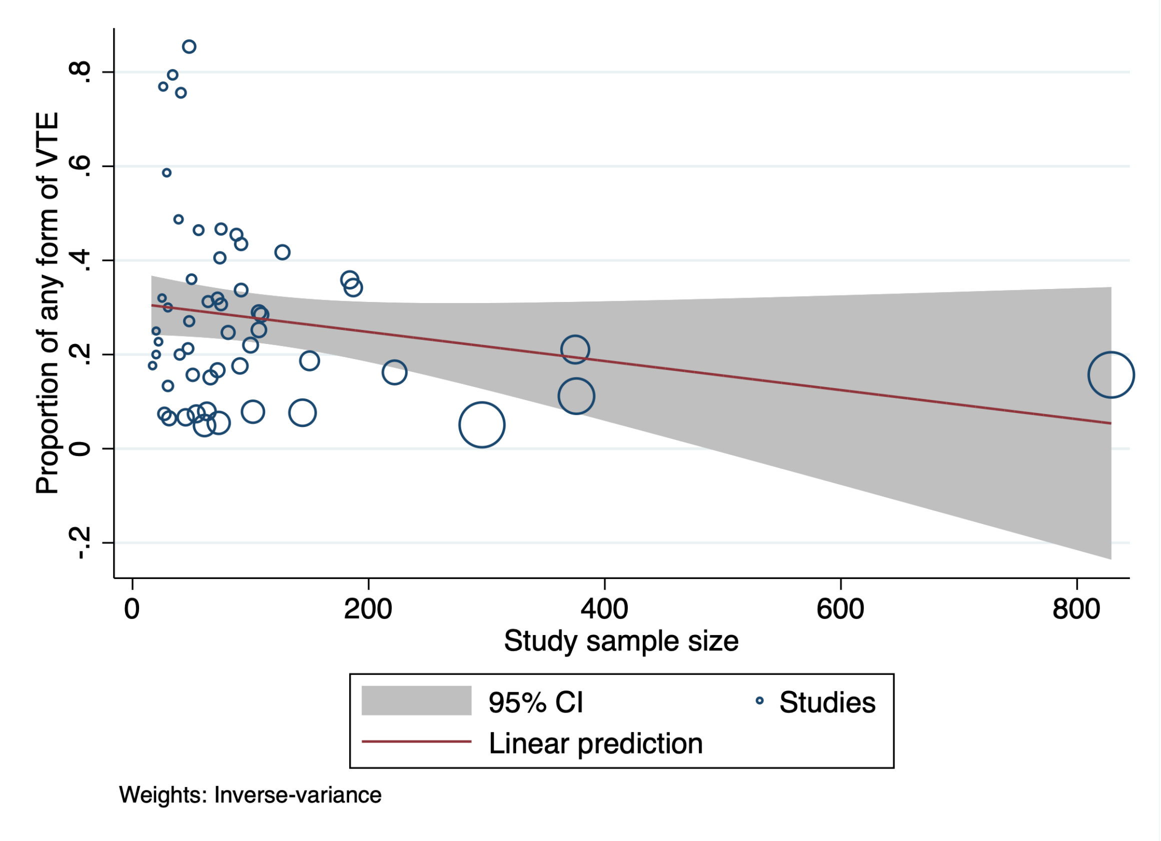

Supplementary figure S6: Correlation between date of publication and VTE incidence

Fig. S6 shows the absence of a correlation between date of publication and reported VTE incidence.

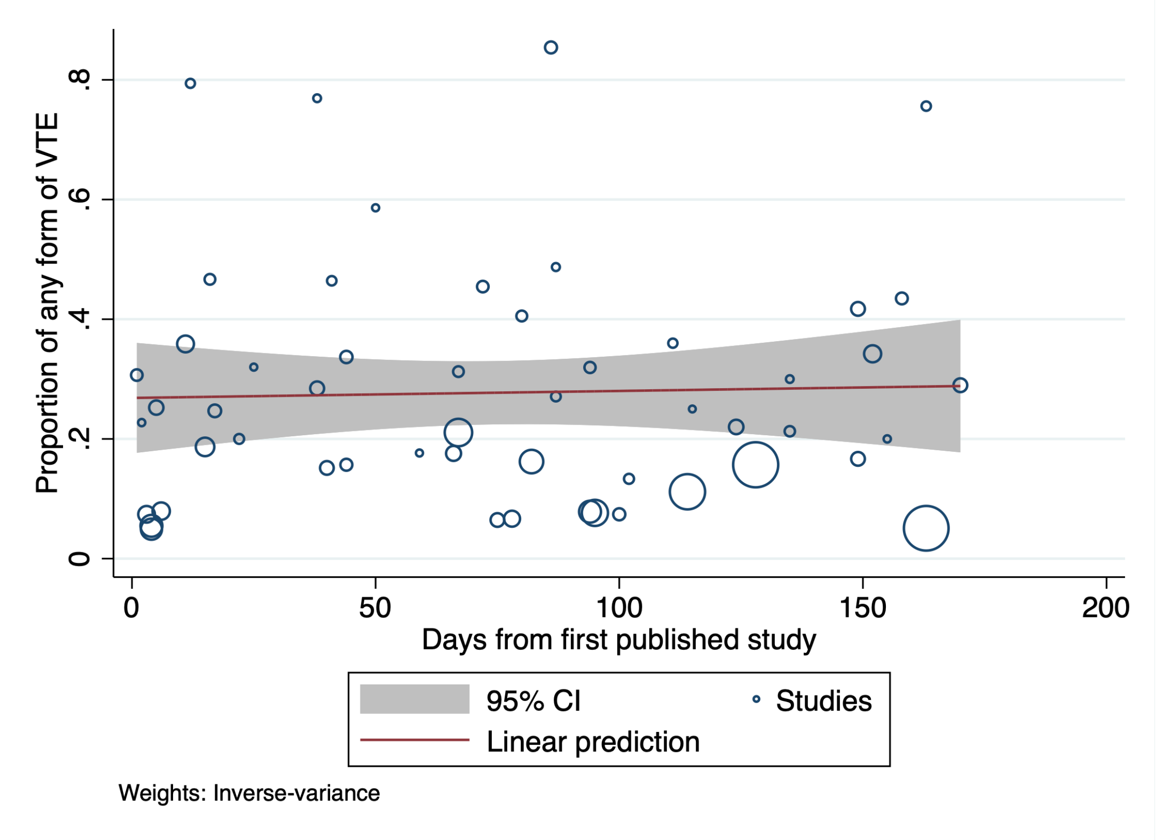

Supplementary figure S7: VTE incidence according to anticoagulant regimens

Fig. S7 shows the incidence of the composite outcome VTE (DVT + PE) according to different anticoagulant regimens.

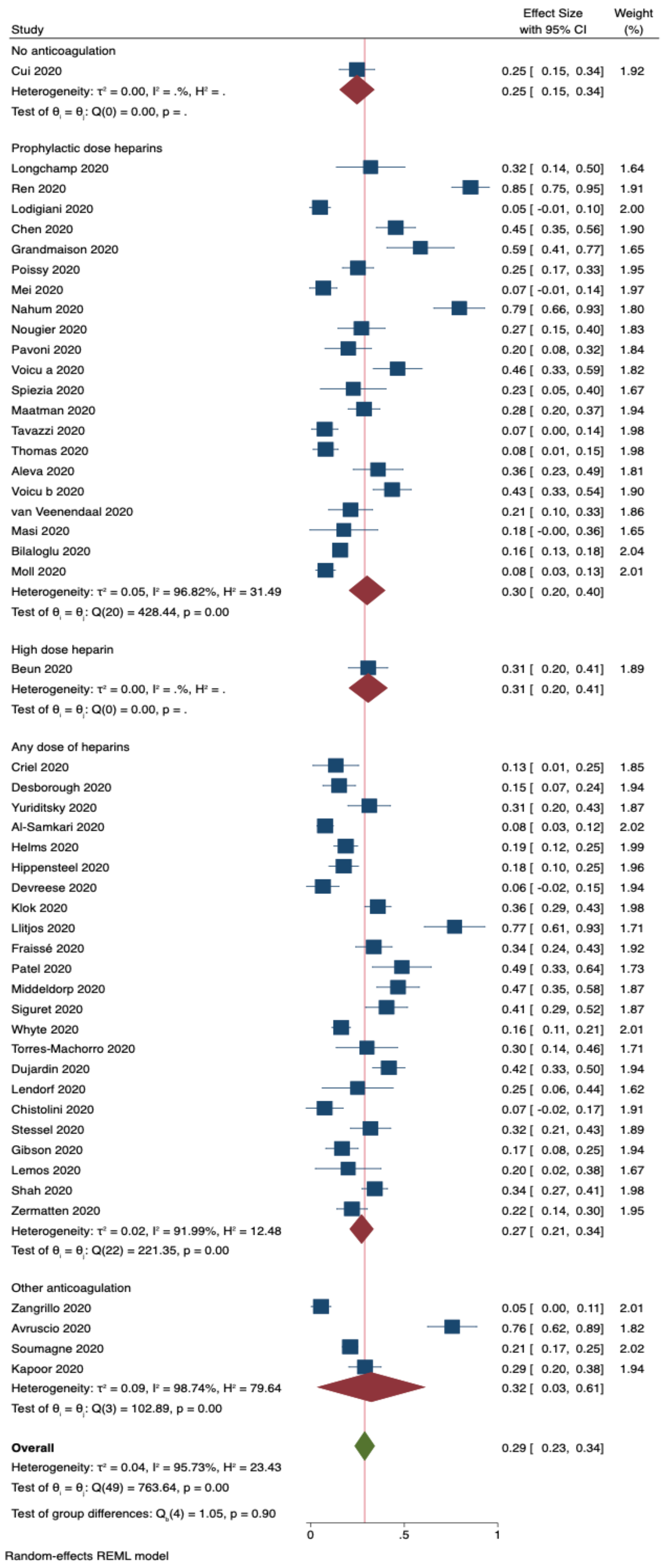

Supplement: Additional File 3 — Supplementary Figures 1–7 show additional subgroup analyses and correlations performed for secondary outcome parameters. [file Data_Sheet_3.PDF]
